# Outbreak of foot and mouth disease and peste des petits ruminants in sheep flock imported for immediate slaughter in Riyadh

**DOI:** 10.14202/vetworld.2017.238-243

**Published:** 2017-02-22

**Authors:** M. A. Mahmoud, S. A. Galbat

**Affiliations:** 1 Department of Parasitology and Animal Diseases, Division of Veterinary Research, National Research Centre, 12622 Dokki, Giza, Egypt; 2 Department of Animal Medicine, Assiut University, Faculty of Veterinary Medicine, New Valley Branch, New Valley Governorate, Egypt

**Keywords:** foot and mouth disease, peste des petits ruminants, Marino sheep, virus, Kingdom of Saudi Arabia

## Abstract

**Aim:**

To detect and identify the causative agent or agents of the following clinical symptoms which were fever, lack of appetite, salivation, vesiculation, erosions of the buccal mucosa, nose, and feet. The signs vary from mild to severe. The mortality rate of the disease is high. The morbidity rate reaches up to 100%. Sheep also show bloody diarrhea and rapid respiration. Sheep flock resident in El-Kharje Governorate.

**Materials and Methods:**

A total of 50 serum samples and 50 buffy coat samples were collected from Marino sheep flock suffered from high mortalities, fever, lameness, diarrhea, stomatitis, and respiratory distress. PrioCHECK^®^ foot and mouth disease virus (FMDV) nonstructural (NS) (marketable enzyme-linked immunosorbent assay [ELISA] kit) was used for revealing of the NS antibodies and liquid phase blocking enzyme immunoassay (LPBE) for identifying the FMD serotype and examined by competitive ELISA (cELISA) for detection of peste des petits ruminants (PPR) antibodies. The buffy coat samples were examined by immunocapture ELISA (Ic ELISA) for detection of PPR antigen.

**Results:**

Using PrioCHECK^®^ FMDV NS: Commercial ELISA kit: 38/50 (76%) of the serum samples were positive for the presence of FMD NS viral proteins. In addition, using LPBE the positive samples were identified as FMD serotype O. Examination of the serum sample by cELISA for detection of PPR antibodies gave positive results in 32/50 (64%). While the Ic ELISA identified 32 (64%) positive reactors for PPR antigen.

**Conclusion:**

This study reflected high susceptibility of the imported sheep flocks to the infection with FMD and PPR viruses, which are endemic in the Kingdom of Saudi Arabia (KSA). Hence, the imported flocks that prepared for slaughter must be vaccinated with the used vaccine in KSA in the quarantine for the control of FMD especially when importation occurs from counters that are free from these diseases.

## Introduction

Foot and mouth disease (FMD) virus or aphthous fever virus is a member of family *Picornaviridae* [1]. It is a highly contagious disease of both wildlife and domesticated even-toed animals. Authors confined more than 65 wild animal species that are susceptible to FMD infection [2]. The role of wildlife in maintaining and dissemination of the FMD virus to other susceptible wild or domestic ruminants is significant [3]. The causative agent of FMD is a positive, single-stranded RNA virus [4].

Serologically, there are seven known serotypes of the virus known as O, A, Asia 1, C, SAT 1, SAT 2, and SAT 3 [5]. At this time, among 178 member states of OIE, 66 recorded countries are FMD free (65 with no vaccination, 1 with vaccination), 10 countries contain FMD free zones. North America, Majority of South America, Western of Europe, Australia, New Zealand and most Island countries in pacific are free of the disease. The other reported immediate outbreak notification to OIE. The most prevalent serotype in Kingdom of Saudi Arabia (KSA) is type O although other serotypes (namely, A, C, Asia-1) are present [4]. The causative agent is excreted in all discharges and secretions of the diseased animals so the virus spreads effectively. Infection occurs through direct and indirect contact with the infected materials [6]. FMD serotype Asia 1 is now constantly present in Asia, together with those of serotypes O and A, frequently in the MiddleEast and irregularly in Europe [7]. Airborne infection can occur for a distant of 10 km and this makes complexity in the disease control [8]. Infection with any of the serotype does not give protective immunity against another [9].

Diagnostic vesicles and erosive changes occur in the mouth, nose, nipples, and feet. The signs vary from mild to severe, while adult animals generally get better; high mortality in young animals is common [10]. Fever, lack of appetite, salivation, vesiculation, erosions of the buccal mucosa, skin of the interdigital spaces, and coronary bands are characteristics [11,12]. The mortality rate of the disease is 5% in adult animals and the morbidity rate of FMD reaches up to 100%. The disease is responsible for serious production losses expressed in low milk production and weight loss due to loss of appetite (vesicles in the mouth). FMD has a huge impact on the trading of animal and animal products [13]. In addition to the death of young animals due to the destruction of heart muscles, high costs for curing infected animals those cannot be sold because of emaciation and/or infertile [6].

FMD virus (FMDV) is a small RNA naked virus with a genome of 8.5 kb. This genome encodes to structural and nonstructural proteins (NSPs). The genome contains a single open reading frame and encodes a poly protein, which is cleaved by viral protease 3C to yield four structural (VP1, VP2, VP3, and VP4) and 10 NSPs (L, 2A, 2B, 2C, 3A, 3B1, 3B2, 3B3, 3C, and 3D) [14].

Antibodies to both structural and NSPs are detected in infected animals. Whereas antibodies to the NSPs could not be detected in vaccinated animals as we use dead vaccine [15]. In FMD diseased animals, antibodies intended to both the SP and NSPs are present. While vaccinated animal produces humoral immunity to the structural protein only, consequently, in using examinations that can detect antibodies against NPS we can discriminate diseased animals from vaccinated one and this what can we obtain using the PrioCHECK^®^ (Prionics AG, Switzerland) FMDV [16].

The control of FMD in KSA depended on restricting animal movement from and to the foci of infection, application of ring vaccination extending from the margin to the center of infection in a radius of 5-10 km, complete disinfection of infected premises and treatment of the diseased animals [17]. The used vaccine in KSA is of two types, polyvalent vaccine (produced by Merial company) which is using for vaccination of cattle and monovalent vaccine (Agrovet Russian company) is using for vaccination of small ruminants.

Peste des Petits ruminants (PPR) has a noteworthy obstacle to the animal industry as it causes separate financial misfortunes in sheep and goats farms [18]. PPR virus (PPRV) affected small ruminants (goats and sheep) and induces gastrointestinal inflammation and bronchopneumonia, so the disease is called in some areas pneumoenteritis [19]. The PPRs virus is a member of genus morbillivirus, *Paramyxoviridae* [20]. This genus includes a group of close antigenicity viruses; measles virus, cattle plague virus, and canine distemper [21].

PPR genome is 15,948 nucleotides long and contains six genes encoding six major polypeptides named as nucleocapsid protein (N), phosphoprotein (P), matrix protein (M), fusion (F) protein, hemagglutinin (H), and large RNA-dependent polymerase protein. The virus is single stranded, negative sense nonsegmented RNA. By partial amino acid sequencing of the (F) protein, all the viral isolates of PPR were classified into four lineages (I, II, III, and IV) [22]. Lineages III and IV were recorded in Saudi Arabia [22]. PPR is endemic mainly in Africa in addition to the Middle East and the Indian subcontinent [23]. The PPR viral particles are execrated in all affected animal secretions and discharges [24]. Transmission of the disease occurs via droplets infection of infected animals [25].

PPR is a transboundary disease, so it was registered in the Middle East and in Arabian Peninsula in such countries as Iran, Iraq, occupied Palestine, Jordan, Kuwait, Lebanon, Oman, Saudi Arabia, United Arab Emirates, Yemen and there is serological prove of the disease in Syria and Turkey in the period between 1993 and 1995 [26]. Saudi Arabia is considered as the major importer of livestock, PPR was first recorded in KSA in 1990 [27], and later on the disease was reported in Eastern central region of the kingdom [28]. The disease characterized by high morbidity rate reach to 100% and in the severe outbreak, mortality can cover the total number of the flock, which perishes economic impact in the developing countries [29]. The control program for PPR depends on quarantine and restrictions on movement of small ruminants to and from affected areas, proper disposal of carcasses (burned or buried), disinfecting the fomites and the application of ring vaccination in the surrounding areas of outbreaks. This vaccine is produced by the national center for veterinary vaccine production in Riyadh.

This study aimed to detect and identify the causative agent or agents of the following clinical symptoms which were fever, lack of appetite, salivation, vesiculation, erosions of the buccal mucosa, nose, and feet. The signs vary from mild to severe. The mortality rate of the disease is high. The morbidity rate reaches up to 100%. Sheep also show bloody diarrhea and rapid respiration. Sheep flock resident in El-Kharje Governorate.

## Materials and Methods

### Ethical approval

International Animal Ethics Committee and local laws and regulations were considered in applying our experiments.

### Animals

This study was performed on a sheep farm resident in El Kharj Governorate in KSA. The farm contained 3000 male Marino sheep imported in April 2012 for immediate slaughter from Uruguay. The sheep quarantined and approved that it is free from both FMD and PPR. Instead of going to the slaughterhouse directly, the owner passed 1000 animals to the markets in Riyadh. The rest of the consignment was sent to his farm in El Kharj. The first part was not sold after mixing with the other animals in the markets. The owner gathered all the animals again on the farm. 3 days passed and the clinical signs began to appear.

**Figure-1:**
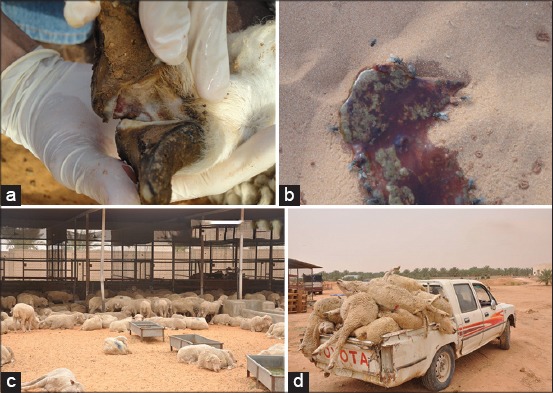
Different clinical signs of foot and mouth disease and peste des petits ruminants diseases.

The symptoms were fever, stomatitis, difficult breathing, salivation, conjunctivitis, lameness, bloody diarrhea, recumbency, and death. The animals suffered from high mortality rate reached to 43.3% (1300 heads) within 2 weeks whereas the morbidity rate was over 90% (Figure-1). The animals under the study were not vaccinated against both FMD and PPR in the KSA. The country of the animal origin is free from FMD and PPR.

### Sampling

#### Blood and serum preparation

A total of 50 blood samples were collected by jugular vein puncture using vacuum tubes containing ethylenediaminetetraacetic acid and other tubes free from anticoagulant. Plane tubes were left to clot. We collected the serum samples aseptically in 2 ml tube and stored it at −20°C until used [30].

The samples were collected from the animals that showing clear clinical signs in the form of fever, stomatitis, difficult breathing, salivation, conjunctivitis, lameness, bloody diarrhea, and recumbency in addition to apparently normal animals.

Buffy coat samples were prepared by centrifugation of the anticoagulated blood tubes at 2000 rpm for 10 min. The buffy coats were collected carefully and separately from the surface of the packed red blood cells in 2 ml tube. The collected buffy coat samples stored at −20°C till used [31].

### Detection of FMD NSP antibodies

The PrioCHECK^®^ FMDV NS: Commercial enzyme-linked immunosorbent assay (ELISA) kit which produced by Prionics Lelystad B.V. to detect antibodies that release due to the infection with the FMD in the sera of the ruminants, camel, and pigs against the viral NSPs. We followed the manufacturer instructions. Test plates were coated with FMDV NSP. 3ABC specific monoclonal antibodies (mAbs) were enclosed in the kit to bind to NSP. The samples were dispensed into the wells of the test plate. Incubation for 1 h at 37°C then washing 3 times is applied. Conjugated mAb with horseradish peroxidase (mAb-HRPO) was added. If the tested serum contains antibodies specific to the NSP, the binding site for the mAb-HRPO will block. After incubation and washing of the plate, the chromogen (tetramethylbenzidine) substrate is dispensed and incubation at room temperature then stopping of the reaction. At a wavelength of 450 nm, the developed color was measured optically. The percentage of inhibition (PI) was calculated as follows:

PI = 100 − (OD test sample/OD 450 negative) × 100.

Sera with PI >50% were scored as positive [32].

### Liquid phase blocking enzyme immunoassay (LPBE) for typing of FMD

Commercial LPBE kit produced by FMD World Reference Laboratory (WRL), Pirbright, UK, was used for detection of antibodies to FMDV. LPBE technique was developed according to Hamblin *et al*. [33]. The LPBE was applied according to standard operating procedure supplied with the kit. Briefly, the test is based on specific blocking of liquid phase FMD antigen by antibodies in the test serum sample. ELISA plates are coated with anti-FMD antibody. Sera premixed with different serotypes of FMD antigens are then added to the coated plates. If specific antibodies are present in the test sera, they will block the antigen and prevent it from binding to the coating antibody so no color appears. If there are no specific antibodies in the tested sera then the antigen will be available to be trapped on the plate, this will be detected by a positive color indicating negative test results.

### Competitive ELISA (cELISA) for diagnosis of PPR

cELISA kit and its protocol provided by IAH (Pirbright Laboratory, UK). The test is depending on the rivalry between the monoclonal antibody against and in the tested serum antibodies for fastening to the H protein antigen [34]. The existence of antibodies in the tested serum sample will prevent the monoclonal antibody (MAb) binding leading to the diminution of expected coloration after the addition of the anti-mouse conjugate and substrate-chromogen solution. The manufacturers also recommended the 50% competition cut-off as the positive value for routine testing. Both the negative and positive cut-off values were utilized from the controls of the test procedure. Using Immunoskan reader produced by Flow Laboratories, UK, we read the ELISA plates at 492 nm wavelength filter. Calculation of the result was gotten automatically by the aid of installed software on a computer connected to the reader. This software is produced by FAO/IAEA, Vienna, Austria, and facilitates getting the PI values directly. The program converts the optical density (OD) values to percentage inhibition via this formula:


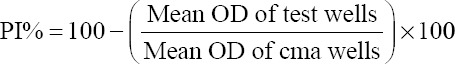


Where, OD represents the optical density value and cma points to the MAb control. Inhibition values more than 50% were considered positive.

### Immunocapture ELISA (Ic ELISA) for detection of PPR antigen

The collected buffy coat samples were examined by the Ic ELISA kit. This kit is capable of detecting the PPR antigen in the buffy coat, nasal swabs, and tissue samples of the supposed animal. The examination was conducted as in accordance with what he has done by Libeau *et al.*, [35] in the Central Veterinary Diagnostic Lab., Ministry of Agriculture, KSA. WRL of Rinderpest and PPR (WRLR/PPR), at Pirbright, UK, supplied the kit and ELISA plates. If the samples contain PPR antigen it attached to the diluted capture antibody that was binded to the plate. On adding the diluted detection antibody, it well binds to the PPR antigen if present in the samples. After addition of the diluted anti-mouse conjugate, a freshly prepared OPD substrate is added and the plate was incubated for 10-20 min at 37°C then addition of the stopping solution. The plate was read in an ELISA plate reader at 492 nm wavelength filter. Calculation of the result was gotten automatically by the aid of installed software on connected computer to the reader. This software is produced by FAO/IAEA, Vienna, Austria, and facilitates the identification of the percentage of positive (PP) values. The OD values were transformed to PP by the next formula:

PP = [100 − (OD control/test sample)] ÷ [Median OD of PPR ref. antigen] × 100

Samples showed PP >18% were considered as positive.

## Results and Discussion

FMD spread rapidly and causes severe financial loss, so it is fundamental to utilize a recommended very sensitive and precise test for untimely diagnosis of the disease in integration to identify the causative serotype or serotypes involved in the outbreak [2].

Despite widespread of FMD virus across the world, but there are some countries that are not registered any cases for the disease since decades. Uruguay is one of these countries that are free from FMD, as well as PPRs disease since 1996 until now. This country is free from PPR and FMD. The control program of FMD in Uruguay depends on vaccination. PPR disease was never been reported in Uruguay [36].

The characteristic epidemiological situation of Uruguay encouraged Arab importers to import sheep from it because of the freedom from FMD and PPR. They are importing sheep for immediate slaughter in Saudi Arabia. Once sheep arrive, they are quarantined, examined to be sure that there are no antibodies to FMD or PPR and free from any other infectious diseases.

In our study, the importer after the release of the sheep from quarantine and prove they are free of FMD and PPR. Instead of sending sheep for immediate slaughter, the importer sent one-third of the consignment to sheep markets in Riyadh and Al-Ahsa and the rest of the consignment was sent to his private farm in Al-Kharj. Sheep prepared for sale is not sold and were returned to the farm after mixing with native sheep. A week after the collection of the consignment fully in his farm, symptoms, and mortality began to appear.

Using the commercial ELISA kit for detection of FMD NSPs antibodies (The PrioCHECK^®^ FMDV NS), 38 samples were positive. Positive samples represent 67% of the total number of samples. The positive sera were examined by LPBE to find out the viral serotype of the virus. The detected viral serotype of FMD was serotype O only (Table-1). This is the predominant serotype of the FMDV in Saudi Arabia [37].

**Table-1:** Results of the tested samples with four different ELISA types for the detection of FMD antibodies and serotyping in addition to the detection of both PPR antibodies and antigen.

50 samples	FMD			PPR			Mixed infection
	
	NSP (%)		LPBE	cELISA (%)		Ic ELISA (%)	
Positive	38/50 (76)		Serotype O	32/50 (64)		18/50 (36)	17/50 (54)
Negative	12 (24)			18 (36)		32 (64)	

FMD=Foot and mouth disease, PPR=Peste des petits ruminants, NSP=Nonstructural proteins, LPBE=Liquid phase blocking enzyme immunoassay, cELISA=Competitive enzyme-linked immunosorbent assay, Ic ELISA=Immunocapture enzyme-linked immunosorbent assay

It is of great benefit to use the LPBE in applying FMD control programs. The test is rapid so we can get the results within the same day.

The method is easy to carry out and does not require extraordinary laboratory conditions like cell culturing.

We used competitive ELISA for in the revealing of the PPRV antibodies in this study due to its elevated specificity (99.8%) and high sensitivity (90.5%) if collated with the standard virus neutralization test [38]. If the serum samples contain specific PPRV antibodies, it will prevent the attachment of the monoclonal antibodies to the coated antigen. Thus, an addition of the chromogen system will not yield a color [39].

Using cELISA for diagnosis of PPR to detect the presence of antibodies against the PPRV, it was found that 32/50 (64%) of the collected sera were positive (Table-1). Prevalence of PPRV infection varied between flocks, ranging from 0.87% to 82.60% and the overall antibody response to PPRV was 22.4% by Singh *et al*. [40]. The variation in the prevalence with that we obtained 64% of sera may be due to the small sample sizes and the tested animals as we tested a herd flock of sheep only.

In our study, we detected the presence of antibodies in sheep actually during the outbreak. Amplified frequency of the disease may be due to the lack of the experience of the animals to these diseases in the country of origin. Furthermore, low age of the animals as all animals are <1-year-old, so it is highly susceptible than the adult. Mixing the animals from different origins facilitate the spreading of the diseases [41]. Many factors increase the animal susceptibility to PPRV. These factors include young age, low maternal immunity intake, the poor nutritional status, and drastic climatic conditions [42].

Using Ic ELISA for detection of PPR antigen in the buffy coat samples, 18/50 (36%) of the samples showed positive results (Table-1). The prevalence of PPR antibodies in the serum samples of the same animals was 64%. The variation in the detection of PPR antigen and antibodies in the same animal samples was expected and reasonable as the antibodies actually remain for a longer periods in the serum than the virus which depends on the presence of the viremic phase. The time of sampling, play a role in detecting the PPRV in the blood [41].

On using the cELISA, we can verify infected from the uninfected animals within the animal population. The presence of antibodies in the serum samples is only due to active infection, not immunization because the animals come from a country prevents vaccination against FMD or PPR. The presence of antibodies to the two diseases is an indication to the incidence of infection in the Kingdom. It has been proven that there are no antibodies in sheep blood before it was allowed to get out of the quarantine.

There are 17/50 sheep revealed mixed infection with both FMD and PPR and this represents 34% of the tested animals. These animals showed antibodies to FMD serotype (O) and PPR in addition to the PPRV antigen (Table-1).

## Conclusion

This study reflected high susceptibility of the imported sheep flocks to the infection with FMD and PPRVs, which are endemic in the KSA. Hence, the imported flocks that prepared for slaughter must be vaccinated with the used vaccine in KAS for the control of FMD and PPR in the quarantine especially when importation occurs from counters that are free from these diseases.

## Authors’ Contributions

MMA and SAG conceived the study performed the fieldwork, collected the samples, carried out the laboratory work, and analyzed the data. MMA drafted and reviewed the manuscript. All authors read and approved the final manuscript.
